# A Versatile and Scalable Approach toward Robust Superhydrophobic Porous Materials with Excellent Absorbency and Flame Retardancy

**DOI:** 10.1038/srep31233

**Published:** 2016-08-09

**Authors:** Changping Ruan, Mengxia Shen, Xiaoyan Ren, Kelong Ai, Lehui Lu

**Affiliations:** 1 State Key Laboratory of Electroanalytical Chemistry, Changchun Institute of Applied Chemistry, Chinese Academy of Sciences, Changchun, 130022, P. R. China; 2 University of Chinese Academy of Sciences, Beijing, 100039, P. R. China

## Abstract

The frequent oil spillages and the industrial discharge of organic contaminants have not only created severe environmental and ecological crises, but also cause a risk of fire and explosion. These environmental and safety issues emphasize the urgent need for materials that possess superior sorption capability and less flammability and thus can effectively and safely clean up the floating oils and water-insoluble organic compounds. Here we present the successful hydrophobic modification of the flame retardant melamine sponge with a commercial fluorosilicone, by using a facile one-step solvent-free approach and demonstrate that the resultant superhydrophobic sponge not only exhibits extraordinary absorption efficiency (including high capacity, superior selectivity, good recyclability, and simple recycling routes), but also retains excellent flame retardancy and robust stability. In comparison to conventional methods, which usually utilize massive organic solvents, the present approach does not involve any complicated process or sophisticated equipment nor generates any waste liquids, and thus is a more labor-saving, environment-friendly, energy-efficient and cost-effective strategy for the hydrophobic modification. Taking into account the critical role of hydrophobic porous materials, especially in the field of environmental remediation, the approach presented herein would be highly valuable for environmental remediation and industrial applications.

The frequent oil spillages and the industrial discharge of organic contaminants have created severe environmental and ecological crises^1^,^2^,^3^,^4^,^5^. Aside from environmental and ecological damage, the spilled oil and organic compounds also cause a risk of fire and explosion, since crude oil and most organic compounds are highly flammable and extremely dangerous when ignited. These environmental and safety issues emphasize the urgent need for materials that possess superior sorption capability and less flammability and thus can effectively and safely clean up the floating oils and water-insoluble organic compounds^6^. To achieve satisfied sorption performance towards various hydrophobic contaminants, the facile combination of desirable wettability, high porosity and strong capillary action is highly required for an advanced adsorbent^7^,^8^,^9^,^10^. The hydrophobic modification of three-dimensional (3D) polymer materials (natural and synthetic) is extensively used for the preparation of 3D hydrophobic adsorbents^11^,^12^,^13^,^14^,^15^,^16^,^17^,^18^,^19^. In recent years, a great number of 3D hydrophobic adsorbents have been fabricated with this approach and demonstrated superior oil/water separation performance^14^,^15^,^16^,^17^,^18^,^19^. Nevertheless, the syntheses of these materials often involve the use of massive solvents. This will not only generate lots of waste liquids, but also require multistep procedures and complex instrumentation, and thus makes the process laborious, time-consuming, costly, and unfit for large-scale preparation. Therefore, the exploitation of simple and versatile strategies that use less or no solvents for the hydrophobic modification of 3D porous polymers is highly valuable, but very challenging.

During the heating with oil bath (silicone oil), we found that the silicone oil began to release smoke around the heat temperature of 200 °C. The “smoke” was actually linear and cyclic silicone oligomers generated by the cleavage or volatilization of silicone chains. These silicone oligomers could form a conformal layer on the surface of various substrates and subsequently crosslink, to lead to the hydrophobic silicone coatings on the substrates^2^,^20^,^21^. Inspired by this principle, we, for the first time, demonstrated that the hydrophobic modification of polymer materials with a commercial fluorosilicone (poly(3,3,3-trifluoropropyl)methylsiloxane, denoted PTFPMS) could be achieved by using a facile thermal vapour deposition technique. Moreover, hydrophobic coatings containing different functional groups (including methyl, ethyl, or phenyl) could be efficiently generated by choosing the appropriate silicone precursors. Most significantly, we realized the successful hydrophobic modification of the flame retardant melamine sponge (melamine-formaldehyde sponge, denoted MF sponge) with this approach and demonstrated that the resultant superhydrophobic MF sponge not only exhibited extraordinary absorption efficiency (including high capacity, superior selectivity, good recyclability, and simple recycling routes), but also retained excellent flame retardancy and robust stability. In comparison to conventional methods, which usually utilize massive organic compounds as solvents, the present approach does not involve any complicated process or sophisticated equipment nor generates any waste liquids, and is thus a more labor-saving, environment-friendly, energy-efficient and cost-effective strategy for the surface hydrophobic modification.

## Results and Discussions

**Figure 1:**
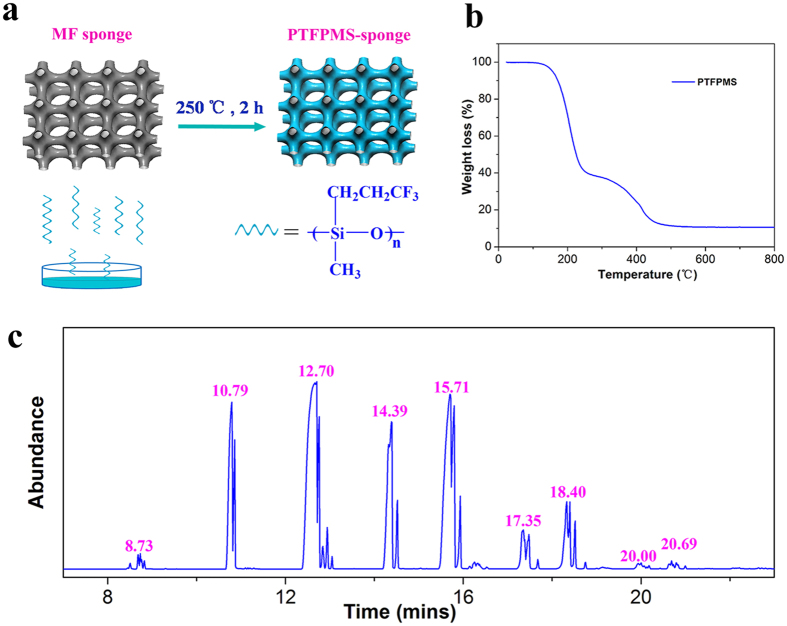
(**a**) Schematic illustration of the synthesis of PTFPMS-sponge; (**b**) TGA of PTFPMS, at a heat rate of 10 °C min^−1^, under air atmosphere; (**c**) Gas chromatogram of PTFPMS.

**Figure 2:**
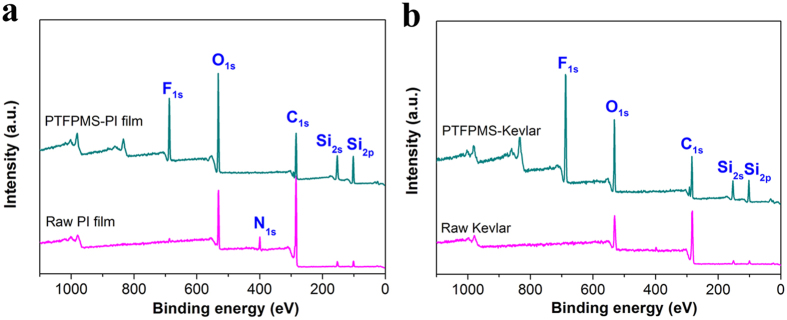
XPS survey spectra of (**a**) PI film and (**b**) Kevlar fibre before and after the coating with PTFPMS.

The success of our approach depends strongly on the gasification temperature of fluorosilicone. As revealed by the thermogravimetric analysis (TGA, Fig. 1b), the PTFPMS began to gasify below the temperature of 150 °C, and nearly 60% of the PTFPMS gasified below the temperature of 250 °C. We carefully optimized the synthetic temperature and time, and found that 250 °C was the optimal temperature (Supplementary Figure S1). Figure 1a illustrates a typical procedure for the hydrophobic modification with PTFPMS by using the thermal vapour deposition technique. Upon heat treatment, the PTFPMS would generate volatiles mainly composed of fluorosilicone short chains with 3–9 monomer units (-Si(CH_3_)(CH_3_CH_2_CF_3_)O-, the constituents of the generated volatiles were investigated by gas chromatography/mass spectrometry (GC-MS) and electrospray ionization/mass spectrometry (ESI-MS), Fig. 1c and Supplementary Figures S2 and S3). These volatile fluorosilicone short chains could form a conformal layer on the surface of substrate and subsequently crosslink, to generate uniform and robust hydrophobic coatings on the substrate. As a proof of concept, we modified two commercial polymers: 2D polyimide film (denoted PI film) and para-aramid fibre (Kevlar, DuPont) with this approach. Expectedly, PTFPMS was successfully coated on the surface of these polymers, as evidenced by the appearance of F and Si signals in corresponding X-ray photoelectron spectroscopy (XPS) spectra (Fig. 2)^22^,^23^.

**Figure 3:**
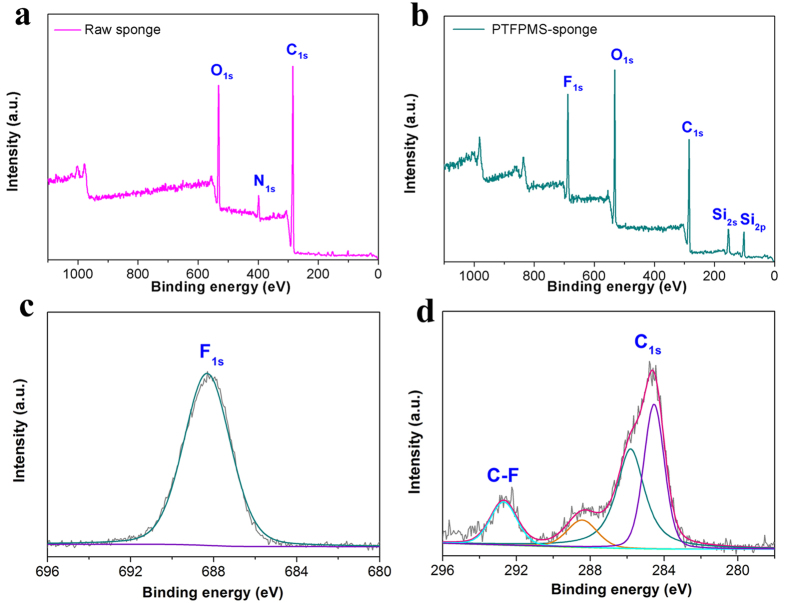
XPS survey spectra of (**a**) raw sponge; (**b**) PTFPMS-sponge after the coating; (**c**) F 1 s and (**d**) C 1 s high-resolution XPS spectra for PTFPMS-sponge.

Encouraged by the successful modification of PI film and Kevlar fibre with PTFPMS, we moved to investigate whether the 3D MF sponge could be modified with PTFPMS by exactly the same approach. In addition to its intrinsic flame-retardant property, the MF sponge is characterized by high porosity, light weight, and robustness, all of which are highly desirable for an advanced absorbent. Yet, the MF sponge is hydrophilic and thus requires hydrophobic modification^24^,^25^,^26^. To boost the applications of MF sponge in the important field of environmental remediation and flame retarding, advanced hydrophobic modification technique that could simplify the production procedures and be compatible with substrates of irregular shape and arbitrary sizes is of great importance. During our previous work, a two-step solvent method was applied for the hydrophobic modification of this MF sponge^6^. In spite of excellent absorption performance and flame retardancy of the resultant sponge, the using of massive solvents (water and ethanol) made the modification process environment-unfriendly, laborious, and costly. Moreover, the modification process of this two-step method was time-consuming (at least 48 hours) and laborious (involved many times of washing), and this was inconvenient for an emergency. The one-step solvent-free approach in this work provided an effective strategy to overcome these limitations. Encouragingly, we found that the MF sponge could indeed be modified with PTFPMS by exactly the same procedure as that of PI film and Kevlar fibre. As evidenced by the XPS spectra in Fig. 3, the disappearance of the signal of N and the presence of the signal of F and Si after the treatment clearly indicated the successful modification of PTFPMS on the surfce. Also, energy-dispersive X-ray spectroscopy (EDS) and Fourier transform infrared (FTIR) spectroscopy confirmed the successful coating of the PTFPMS on the surface of MF sponge (Supplementary Figures S4 and S5). Moreover, EDS mapping images revealed the uniform distribution of F and Si across the whole fiber, indicating the uniformity of the PTFPMS coating (Supplementary Figure S6). In comparison to our previous method, the present approach does not involve any complicated process or the use of any solvent nor generates any waste liquids, and is thus a more labor-saving, environment-friendly, energy-efficient and cost-effective strategy for the surface hydrophobic modification of MF sponge.

**Figure 4:**
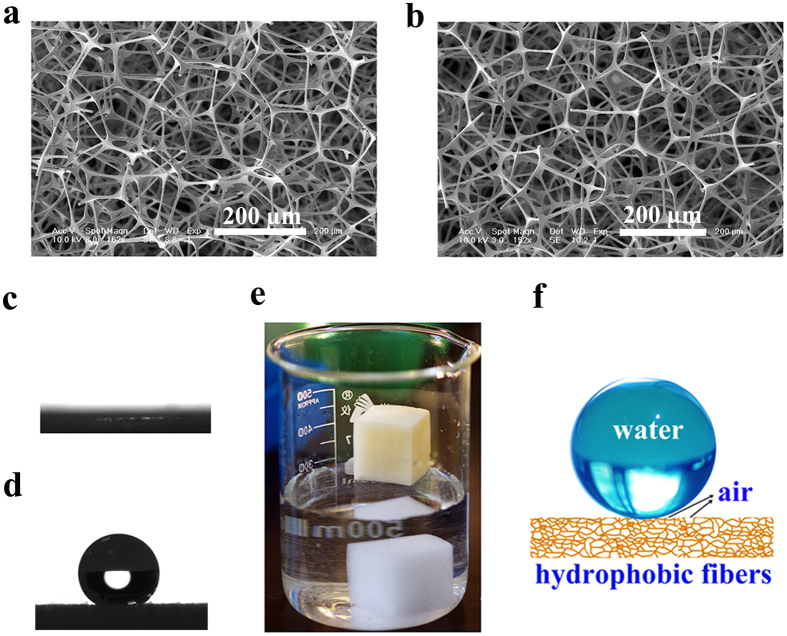
Characterization of PTFPMS-sponge. (**a**) SEM image of the unmodified MF sponge; (**b**) SEM image of PTFPMS-sponge; (**c**) The water contact angle of the unmodified MF sponge; (**d**) The water contact angle of PTFPMS-sponge (158.5 ± 1.62°); (**e**) Photograph of the unmodified MF sponge and the PTFPMS-sponge after placement on the water surface; (**f**) Illustration of the wetting mechanism of water droplet residing on the surface of PTFPMS-sponge.

After modification with PTFPMS, the open porous networks of the sponge were well-preserved, as shown by scanning electron microscopy (SEM) images (Fig. 4a,b). The open porous network and the 3D interconnected pores of the sponge will not only provide channels for mass transfer (both gas and liquid) and capillary action, but also create the desired reservoir for the absorbed liquids, thus ensuring superior adsorption performance, especially in terms of capacity and kinetics^6^,^27^. In addition, the present approach is versatile and could be applicable not only to different substrates but also to various silicones. By choosing the appropriate silicone precursors, hydrophobic coatings containing other functional groups (including methyl, ethyl, or phenyl, see Supplementary Figure S7) could be successfully generated with this approach.

Subsequently, we performed water contact angle measurements to examine the surface wettability of the PTFPMS coated MF sponge (denoted as PTFPMS-sponge). As anticipated, a dramatic change in the surface wettability of MF sponge occurred after the PTFPMS coating. As shown in Fig. 4d, the PTFPMS-sponge demonstrated a high water contact angle of 158.5 ± 1.62°, suggesting its superhydrophobicity and water-repellent nature. In contrast, the water droplet penetrated into the untreated MF sponge immediately after reaching its surface (Fig. 4c), owing to its superhydrophilicity. The superhydrophobicity and water repellency of PTFPMS-sponge were further confirmed by the phenomenon that it floated on water without sinking, after being placed on water (Fig. 4e). In sharp contrast, the uncoated MF sponge sank to the bottom quickly after reaching the water surface, which was ascribed to its open-pore structure and superhydrophilicity. It is well accepted that the surface hydrophobic coating itself is not the sole contributor to the superhydrophobicity of materials. Besides from the material composition, the surface roughness and the fraction of air trapped beneath the liquid are another two important factors that determine the wetting property of surface^2^. In the present case, because of the unique combination of the 3D interconnected network, the high porosity (99.5%), and the hydrophobic coating, the water on the PTFPMS-sponge surface was supported not only by the interconnected hydrophobic fibers of the sponge, but also by the air pockets trapped in the sponge, which thereby yielded a high water contact angle (as illustrated in Fig. 4f).

**Figure 5:**
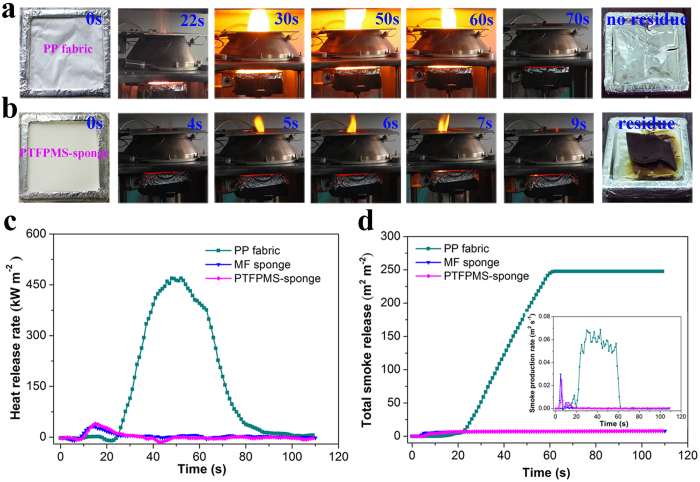
Combustion behavior of PTFPMS-sponge. (**a**) The combustion process of the PP fabric, no residue left after the combustion; (**b**) The combustion process of PTFPMS-sponge; (**c**) HRR curves; (**d**) TSR curves, inset: SPR curves.

Taking into account that the high nitrogen content in PTFPMS-sponge was retained (determined by elemental analysis, 52.2 wt%, even slightly higher than that of the raw MF sponge, 48.7 wt%), we believed that the PTFPMS-sponge was able to inherit the intrinsic flame-retardant nature of MF sponge and thus had the potential of reducing the risk of fire^6^. To verify this speculation, we used cone calorimetry to investigate the combustion behavior of PTFPMS-sponge, especially the heat and the smokes produced during the combustion. It is well-known that the heat and the smokes released during the burning are the major causes of injury and death in the fires^28^,^29^. For comparison, the untreated MF sponge and commercial nonwoven polypropylene fabric (denoted PP fabric, which is one of the most commonly used commercial absorbent for the spilled oils) were also subjected to the same test. After being ignited, the PP fabric gave off a bright and vigorous flame, and it continued to burn for almost 50 seconds without any residue left (Fig. 5a). In contrast, the flame on the PTFPMS-sponge was weak and extinguished within 5 seconds, leaving behind a black brown residue (Fig. 5b). Remarkably, the heat release rate (HRR) of PTFPMS-sponge was much lower than that of PP fabric, similar to that of untreated MF sponge (Fig. 5c). This is indicative of the flame-retardant property of PTFPMS-sponge, implying that this facile modification approach didn’t compromise the flame retardancy of MF substrate. Also, the total smoke release (TSR) curves and smoke production rate (SPR) curves (Fig. 5d) suggested that the PTFPMS-sponge and the untreated MF sponge released significantly less smoke than that of PP fabric, indicating their reduced toxicity during the burning (Fig. 5d)^28^,^29^. Taken together, the PTFPMS-sponge indeed inherited the intrinsic flame-retardant nature of MF sponge, and thus was expected to be used as a flame retardant absorbent for flammable organic compounds.

Given that the mild modification approach would not damage the original structure and mechanical stability of MF substrate, the as-synthesized PTFPMS-sponge should possess excellent mechanical property. As expected, compression tests demonstrated that the PTFPMS-sponge indeed inherited the superior mechanical stability and excellent flexibility of the pristine MF sponge (Supplementary Figures S8, S9, and S10). Impressively, the PTFPMS-sponge retained its 3D interconnected network and high porosity without obvious structural damage even after 1000 cycles of repeated compression/releasing test (50% strain). The excellent mechanical property could enable us to recycle the sponge and recover the absorbed liquids by squeezing the PTFPMS-sponge, and this endowed the as-synthesized sponge with excellent reusability.

In addition, we found that the PTFPMS-sponge also showed superior thermal stability and solvent-resistant, which is advantage for its broad range of applications, long-term durability, and recyclability. After heat treatment of the PTFPMS-sponge at 250 °C for 1 hour at atmosphere, the PTFPMS coating layer and the superhydrophobic behavior of the sponge remained unchanged (Supplementary Figure S11), revealing its superior thermal stability. This originates from the fact that both the MF resin skeletons and the PTFPMS coating layers on the surface possessed strong resistance to high temperature (TGA in Supplementary Figure S12)^6^,^30^. Also, the PTFPMS-sponge was highly stable and robust against various organic compounds. Specifically, its structural integrity and superhydrophobicity survived 12 hours of immersion test (in toluene, cyclohexane, dichloromethane and chloroform, respectively, Supplementary Figures S13, S14, and S15). Even after the boiling treatment in various organic solvents for 1 hour (at the corresponding boiling point of cyclohexane, toluene, and xylene, respectively), the sponge still maintained its 3D interconnected network and superhydrophobic property (Supplementary Figures S16, S17, and S18). In striking contrast to PTFPMS-sponge, the commercial PP fabric showed inferior thermal and chemical stability (Supplementary Figures S19 and S20).

**Figure 6:**
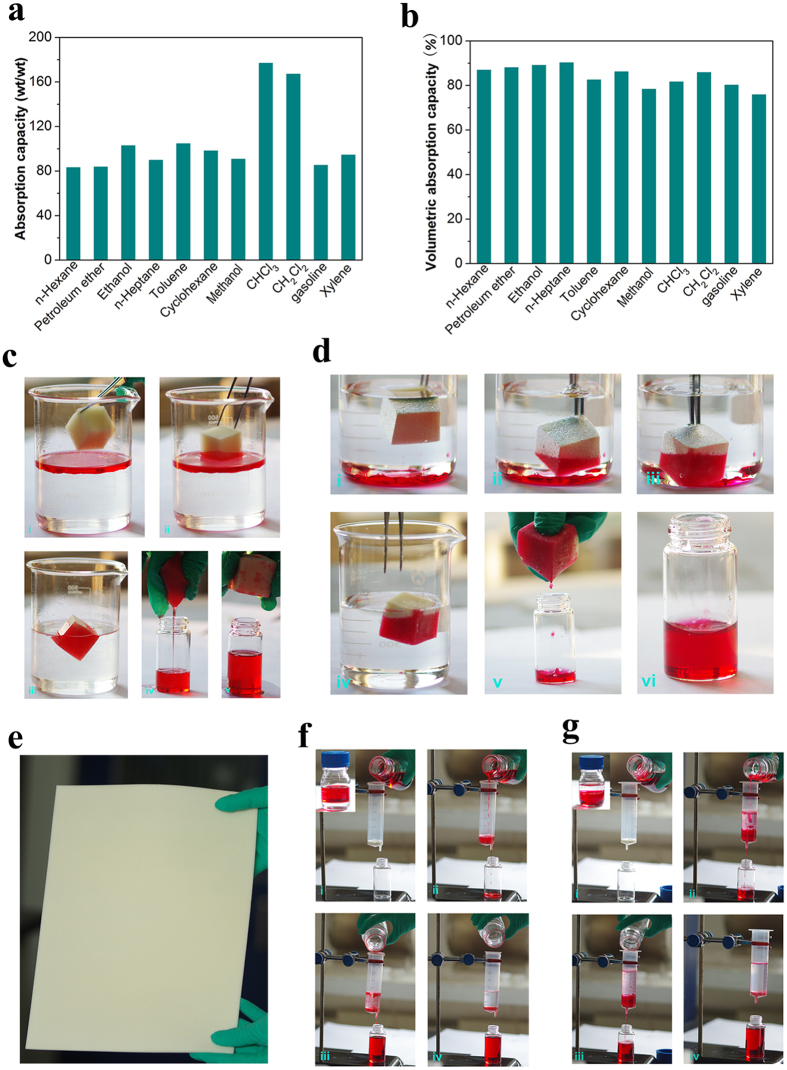
Absorption performance of PTFPMS-sponge. (**a**) Gravimetric absorption capacities towards various organic compounds; (**b**) The corresponding volumetric absorption capacities; (**c**) The absorption and recycling process of n-hexane; (**d**) The absorption and recycling process of the underwater chloroform; (**e**) Photograph of PTFPMS-coated MF membrane; (**f**) The separation of n-hexane/water mixture by filtration; (**g**) The separation of dichloromethane/water mixture by filtration.

Given the successful combination of high porosity, superhydrophobicity, and robust stability, the PTFPMS-sponge could serve as a perfect candidate for the quick removal of various oils and organic solvents. Therefore, the adsorptive property of PTFPMS-sponge was investigated in detail, and the results were summarized in Fig. 6. Remarkably, the PTFPMS-sponge demonstrated excellent absorption capacities towards various organic compounds, up to 83–177 times of its own weight (Fig. 6a). Such high absorption capacities are at least 12–19 times higher than that of commercial PP fabric (4.7–12.4 times of its own weight, Supplementary Figure S21). Furthermore, it is estimated that nearly 75.6–90.3% of the volumetric absorption capacity was achieved, comparable to that of ultralight carbon aerogels (Fig. 6b)^31^,^32^,^33^.

**Figure 7:**
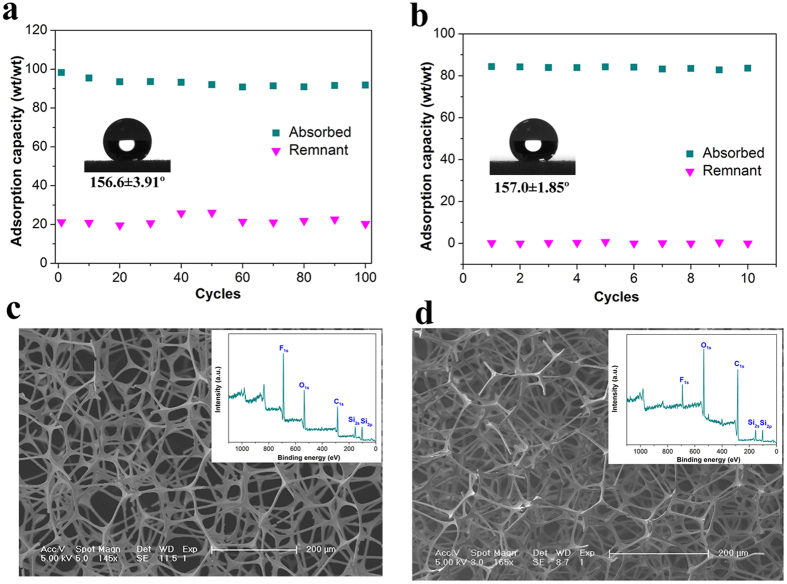
Absorption capacity and residual amount of n-hexane (**a**) over 100 absorption/squeezing cycles and (**b**) over 10 absorption/distillation cycles, Inset: the corresponding water contact angles after cycles. SEM images and XPS survey spectra of PTFPMS-sponge (**c**) after 100 cycles of absorption/squeezing test and (**d**) after 10 cycles of absorption/distillation cycles.

For an advanced absorbent, superior recyclability of the absorbent and good recoverability of the absorbed liquids are other essential requirements^6^,^8^. Gratifyingly, PTFPMS-sponge demonstrated excellent robustness and extraordinary reusability both in terms of manual squeezing and distillation (Fig. 7), which was mainly attributed to its mechanical robustness, thermal stability, durability and tolerance to organic solvents. As shown in Fig. 7a, the sponge still maintained 93.4% of its initial capacity even after 100 repetitions of absorption/squeezing, indicating its superior recyclability. Also, after repeating the adsorption/distillation process for 10 cycles, no obvious adsorption capacity decline of the sponge was observed compared with the first cycle (Fig. 7b).

The outstanding adsorptive property of the PTFPMS-sponge prompted us to survey its feasibility for the separation of oil/water mixtures. As illustrated in Fig. 6c, when brought into contact with the model organic compound (n-hexane, dyed with oil red) floating on water, PTFPMS-sponge quickly absorbed the n-hexane while repelling the water. The process was fast, and the uptake of the entire n-hexane was finished within a few minutes. Such speedy absorption kinetics was mainly ascribed to the unique combination of the oleophilic nature, high porosity, and capillary action of PTFPMS-sponge. After finishing the absorption, the sponge still floated on the water, owing to its superhydrophobic nature and low density, and thus it was easy to be collected to recover the n-hexane. Because of the mechanical robustness and the flexibility of the sponge, the absorbed organic could be recycled easily through mechanical squeezing. In contrast to the floating n-hexane, chloroform sank to the bottom of water because of its higher density than water. Even so, it could still be absorbed and recycled efficiently by the PTFPMS-sponge (Fig. 6d).

The modification technique in the present work was compatible with substrates of different shapes and sizes, PTFPMS-coated MF membrane with the paper-like morphology (Fig. 6e) could thus be prepared with this approach for the installation of simple filtration apparatus. It is well known that membrane filtration is another effective method for the separation of oil/water mixture^34^. Because of the high porosity, the superhydrophobic property, and water-repellent nature of the membrane (as evidenced by its SEM images and high water contact angle in Supplementary Figure S22), it did not allow water but did permit the organics (n-hexane and dichloromethane, Fig. 6f,g) to spread and penetrate through the membrane quickly. Consequently, the oil/water mixture could be effectively separated. Due to excellent durability of the PTFPMS coating, the superhydrophobic membrane could be applied successively and effectively to the selective separation of oil/water mixture.

**Figure 8:**
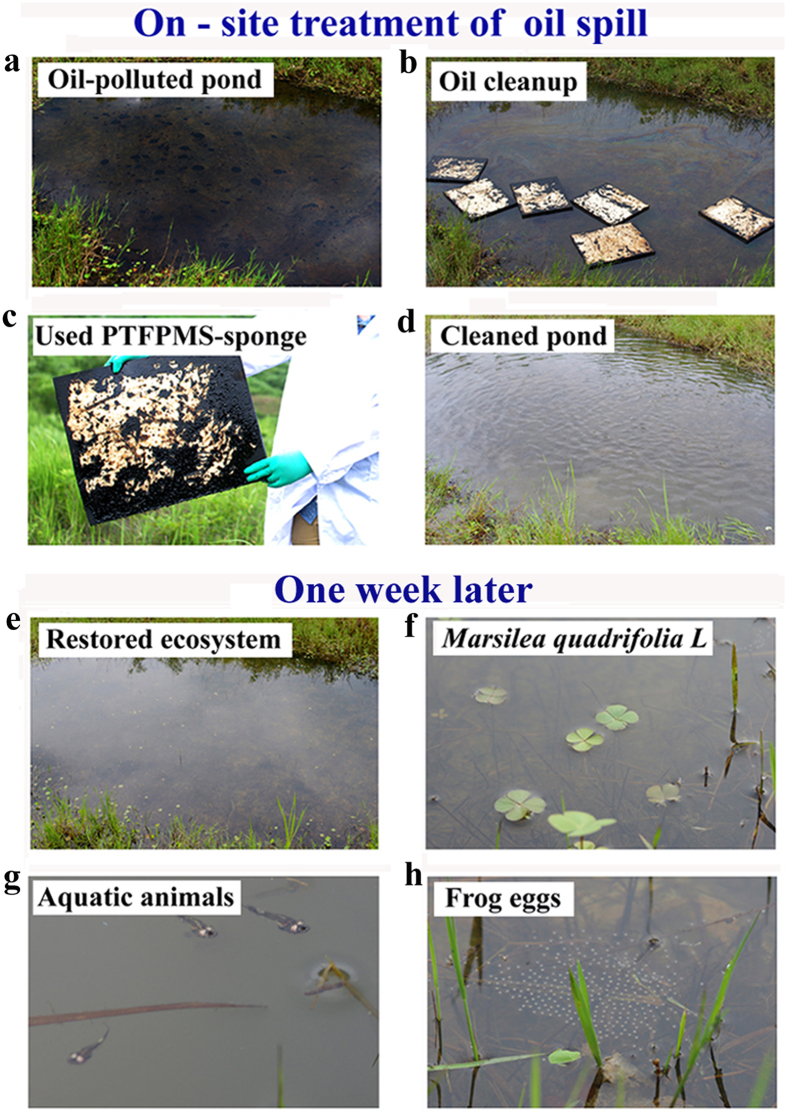
Removal of crude oil from polluted water in a pond. (**a**) water polluted by crude oil; (**b**,**c**) removal of crude oil by using PTFPMS-sponge (size of sponge: 48 cm × 40 cm × 1.5 cm); (**d**) Photograph showing the cleaned water surface after the oil removal; (**e**–**h**) Photographs showing that the aquatic animals and plants began to live in the pond, one week later after the cleanup.

With the low cost of its raw materials and its ease of fabrication, large-scale preparation of the PTFPMS-sponge was realized by simply scaling up the synthesis. The ease of scalability supported our efforts to clean up the spilled oils on a large scale. As shown in Fig. 8, a pond polluted by crude oil was treated by large-sized PTFPMS-sponges to remove the floating oils. After efficiently removing all the oils, the water became clean (Fig. 8d), which was in stark contrast to the black-brown and oily water surface before the treatment. Chemical and biological oxygen demand (COD and BOD, 5-day) measurements were carried out to monitor organic pollutants in the pond before and after the oil cleanup^35^,^36^,^37^,^38^. COD and BOD of the polluted water were 1380 mg L^−1^ and 46.9 mg L^−1^, respectively. Such high values suggested that the water samples were heavily contaminated with organic compounds. After the treatment, COD and BOD of water samples were reduced to 21.7 mg L^−1^ and 5.4 mg L^−1^, respectively. These values are lower than the limits for first grade standard of Integrated Wastewater Discharge Standard (COD < 50 mg L^−1^; BOD < 10 mg L^−1^)^39^,^40^,^41^,^42^. According to the Environmental Quality Standard for Surface Water, COD and BOD values of the treated water is lower than that of water of grade IV (COD ≤ 30 mg L^−1^; BOD ≤ 6 mg L^−1^), which is for industrial and recreational use^41^,^42^. This indicated that the water quality was greatly improved after the treatment with PTFPMS-sponges. Moreover, one week after the treatment, aquatic animals and plants began to live in the pond (Fig. 8e–h). It is well accepted that eggs and larvae of aquatic animals are particularly susceptible to the toxic chemicals in the crude oil^43^,^44^,^45^. Fortunately, frog eggs were observed in the pond (Fig. 8h). These observations further indicated the improved water quality after the removal of oil, and also suggested that the PTFPMS-sponge was an environment-friendly adsorbent.

In summary, an outstanding superhydrophobic sponge with robust stability, remarkable oil-water separation efficiency and flame retardancy has been synthesized through a facile one-step solvent-free process. The avoidance of any solvents in the synthesis will not only reduce the cost of raw materials, but also lead to dramatic reduction of waste production. The synthetic procedure is a simple one-step process without any pre- or post- treatment step, and thus could save lots of time, energy and labor. Also, the ease of fabrication is a great advantage for an emergency, such as an accidental oil spill and a sudden leakage of organic contaminants, since large-scale preparation of PTFPMS-sponge can be achieved easily and quickly. Moreover, the successful introduction of different functional groups with this approach could allow for tailoring the sorption property of various substrates, which is beneficial for their potential use as separation media for diverse target compounds. Taking into account the critical role of hydrophobic porous materials, especially in the field of environmental remediation, the approach presented herein would be highly valuable for environmental remediation and industrial applications.

## Methods and Materials

### Synthesis

In a typical synthesis of the PTFPMS-sponge, 50 ml of PTFPMS (Viscidity: 500–1000, methyl-terminated) was placed into a glass desiccators, several pieces of MF sponge (6–8 mg cm^−3^) were placed above the PTFPMS. The container was covered and heated at 250 °C for 2 hours. After cooling, the obtained PTFPMS-sponge was taken out and used directly. To maintain sufficient hydrophobic coating, excessive amounts of PTFPMS were used each time. PTFPMS-coated MF membrane was also prepared through exactly the same method. In the large-scale production, the reaction vessel was a stainless steel box with the size of 50 cm × 50 cm × 12.5 cm.

### Material characterization

SEM images were performed on a FEI/Philips XL30 ESEM FEG field-emission scanning electron microscopy system. The XPS spectra were collected on a VG ESCALAB MKIIX-ray photoelectron spectrometer using Al-Kα as the exciting source. FTIR spectroscopy was performed on a Bruker Vertex 70 spectrometer. TGA and differential scanning calorimetry (DSC) measurements were performed on a Perkin-Elmer TGA-2 thermogravimetric analyzer from room temperature to 800 °C at a rate of 10 °C min^−1^ under air atmosphere. The water contact angle measurements were carried out on a DSA30 contact-angle system (Kruss, Germany) at ambient temperature. The volume of water droplet was 4 μL. The compression tests were carried out on an Instron universal testing machine with the compressive rate of 50 mm min^−1^. Flammability tests were performed on a Dual Cone Calorimetry (FTT, U.K.), according to ISO5660 Standard. The size of samples was 95 mm × 95 mm × 5 mm. The heat flux was 50 kW m^−2^. Exhaust flow rate was 24 L s^−1^, and the spark was continuous until the sample ignited.

### Oil and organic solvent absorption tests

In the absorption tests, various oils and organic solvents (including n-hexane, ethanol, toluene, cyclohexane, n-heptane, petroleum ether, methanol, chloroform, dichloromethane, xylene, and gasoline) were tested. In a typical test, the absorbent (PTFPMS-sponge and PP fabric, respectively) was dropped into the organic liquids until it was completely filled with the corresponding liquids, and then it was taken out and quickly weighed to avoid evaporation of the organic liquids. The absorption capacity was calculated by *W*_*a*_*/W*_*0*_ *-1*. *W*_*0*_ is the weight of the dry absorbent before sorption, and *W*_*a*_ is the total weight after saturation with corresponding organic liquid. Each oil or organic solvent was tested three times. In the cyclic absorption/squeezing and absorption/distillation measurement, the absorption capacity was obtained through the same method. The total weight of the absorbent and the residual liquid after the squeezing or distillation (*W*_*r*_) was measured again to calculate the remnant capacity (defined as *W*_*r*_*/ W*_*0*_ *-1*). *W*_*0*_ is the weight of the dry absorbent before the first cyclic sorption.

## Additional Information

**How to cite this article**: Ruan, C. *et al*. A Versatile and Scalable Approach toward Robust Superhydrophobic Porous Materials with Excellent Absorbency and Flame Retardancy. *Sci. Rep*. **6**, 31233; doi: 10.1038/srep31233 (2016).

## Supplementary Material

Supplementary Information
