# Supplementary material for: A Versatile and Scalable Approach toward Robust Superhydrophobic Porous Materials with Excellent Absorbency and Flame Retardancy
Source: Sci Rep. 2016 Aug 9;6:31233. doi: 10.1038/srep31233 (PMC4977488; doi:10.1038/srep31233)
Supplement: Supplementary Information [file srep31233-s1.doc]

Supplementary information

Correspondence and requests for materials should be addressed to K.A. (klai@ciac.ac.cn) or L.L (lehuilu@ciac.ac.cn).

**A Versatile and Scalable Approach toward Robust Superhydrophobic Porous Materials with Excellent Absorbency and Flame Retardancy**

Changping Ruan^1,2^, Mengxia Shen^1,2^, Xiaoyan Ren^1^, Kelong Ai^1^* & Lehui Lu^1^*

^1^State Key Laboratory of Electroanalytical Chemistry, Changchun Institute of Applied Chemistry, Chinese Academy of Sciences, Changchun, 130022, P. R. China

^2^University of Chinese Academy of Sciences, Beijing, 100039, P. R. China

C.P.R. and M.X.S. contributed equally to this work.


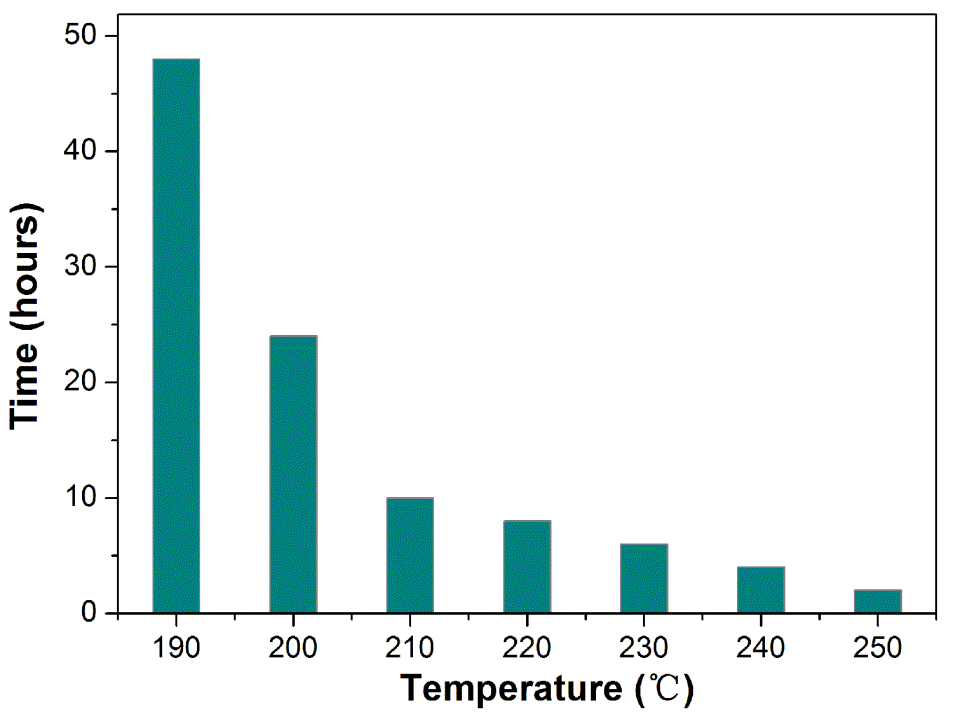


**Figure S1** | To maintain the hydrophobic property, a thin layer of hydrophobic coatings (PTFPMS) able to cover the entire surface of substrate is necessary. TGA result of PTFPMS indicated that the PTFPMS began to gasify below the temperature of 150℃. At the temperature of 190℃, approximately 20% of the PTFPMS gasified. It is worth to mention that nearly 60% of the PTFPMS gasified below the temperature of 250℃. We carefully optimized the experimental condition of the hydrophobic modification. We found that, at relatively lower temperature, a longer period of time was necessary to form sufficient hydrophobic coatings. With increasing the synthetic temperature, the time needed for the synthesis could be reduced. This is reasonable, since more volatile fluorosilicone short chains would be generated at higher temperature.


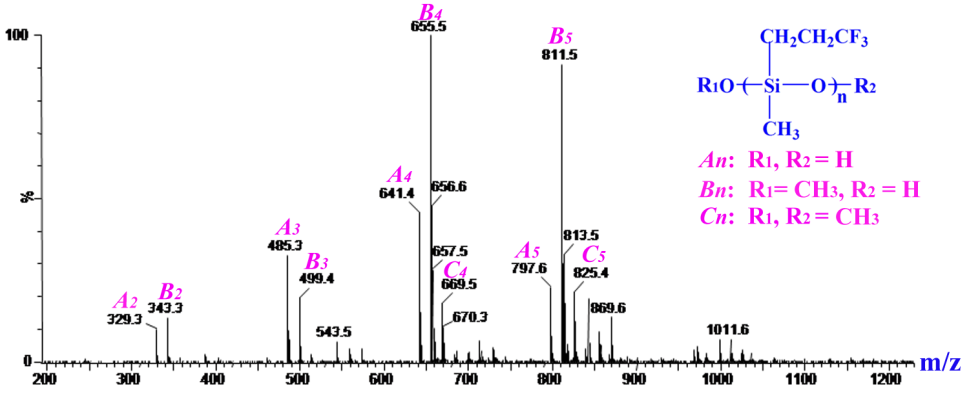


**Figure S2** | Electrospray ionization/mass spectrometry (ESI-MS) analysis of PTFPMS (operated in negative ion mode) and the corresponding identification of the main pseudo-molecular ions (M-1). ESI is a “soft ionization” technique. It is an attractive ionization method for the detection of oligomeric species with little or no fragmentation. Therefore, [M-H]^-^ ions were the mainly species formed and detected in the ESI-MS measurement. The ESI-MS results confirm that the PTFPMS mainly contains linear oligomers. Due to the limitation of ESI-MS method, polymers with higher molecular weight (approximately higher than 1000) is difficult to be detected. Although hydroxyl-terminated and methyl-terminated linear PTFPMS oligomers were both detected during the ESI-MS measurement, the FTIR spectra of PTFPMS (Figure S5) indicated that the hydroxyl group is almost negligible. Such inconsistent results should be mainly attributed to the limitation of ESI ionization method. We speculate that the hydroxyl-terminated polymers are easier to form [M-H]^-^ ions than methyl-terminated polymers.

*
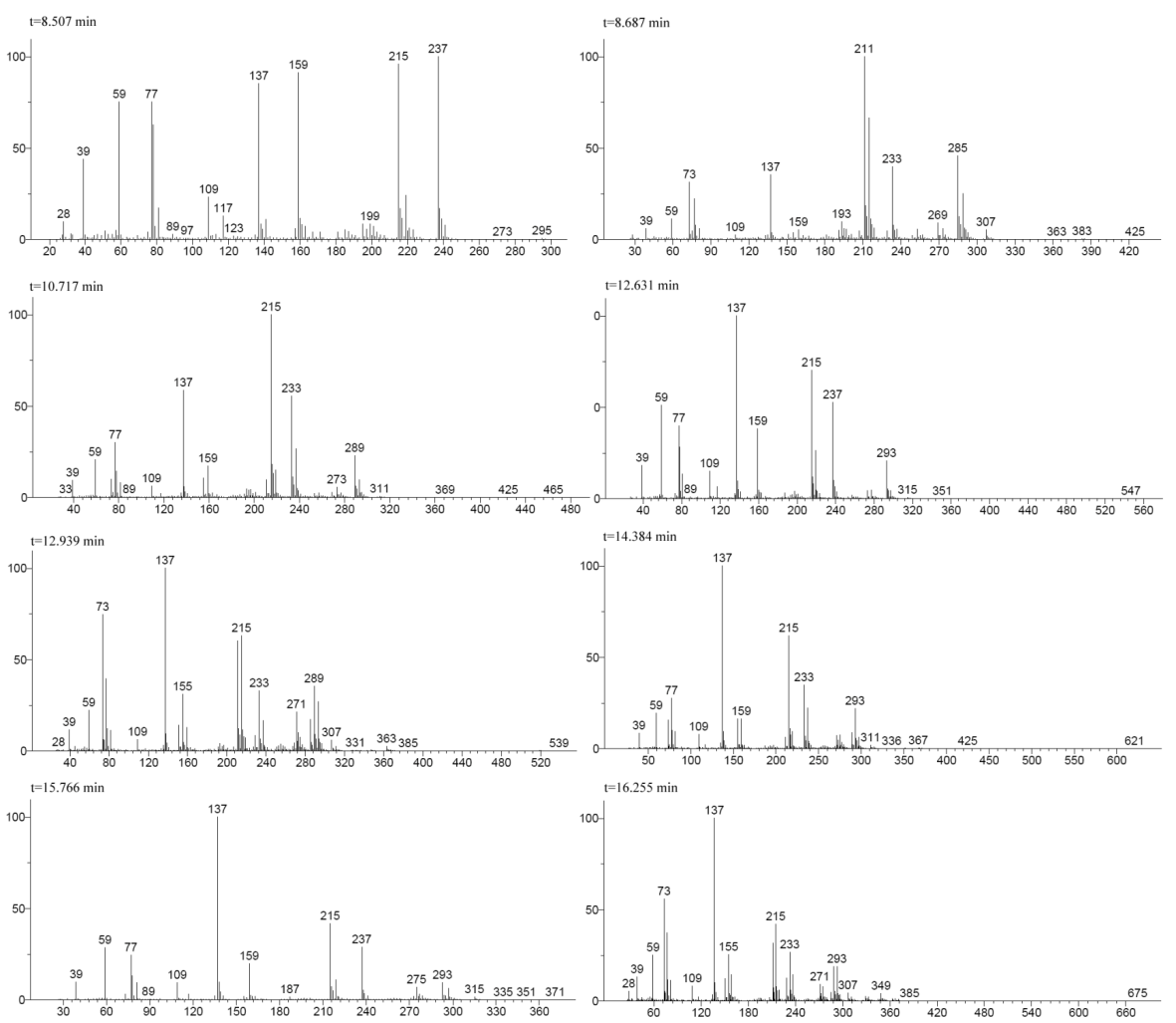
*


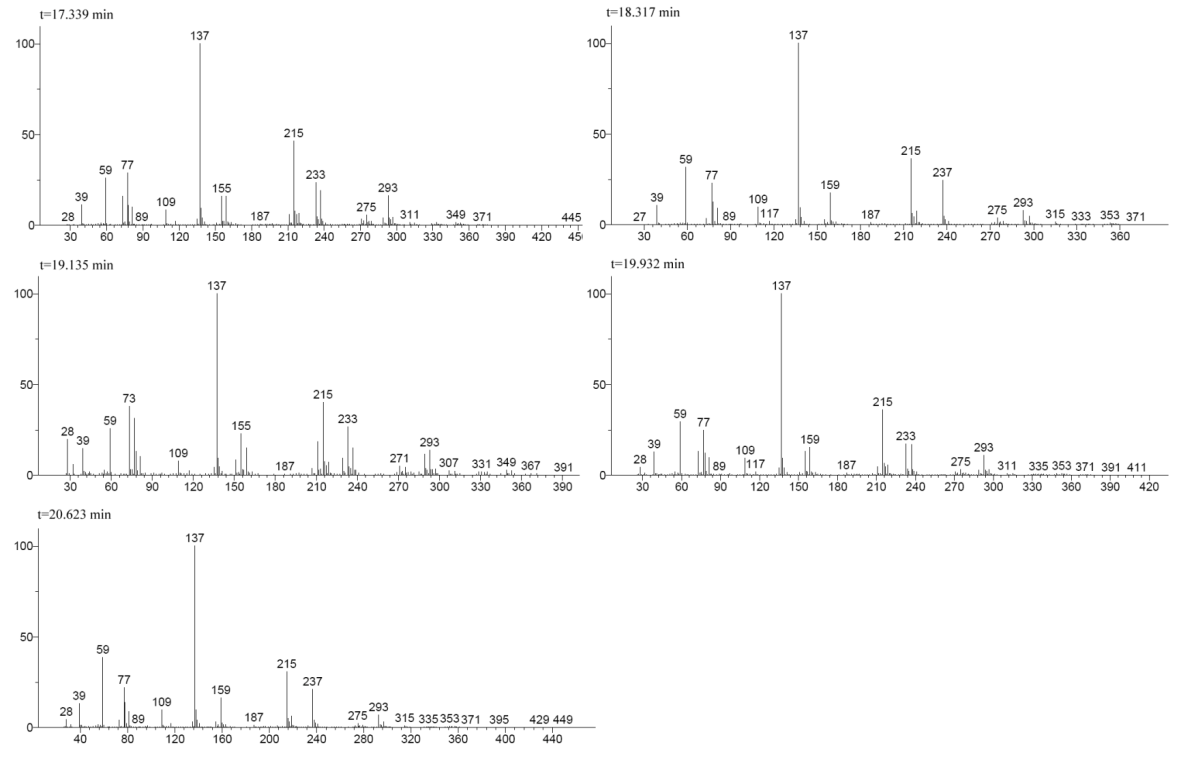


**Figure S3** | The corresponding mass spectrometry (MS) results of PTFPMS during the Gas chromatography-mass spectrometry (GC-MS) measurement. The “hard ionization” technique ([electron ionization](https://en.wikipedia.org/wiki/Electron_ionization) (EI)) will result in the creation of many fragment ions of low mass to charge ratio (m/z), with few or no formation of molecular ions. Therefore, it is difficult to ascertain the exact [molecular](javascript:void(0);) [weight](javascript:void(0);) of each oligomeric species. However, these species eluted at different retention times showed similar patterns of ionized fragments, indicating the fact that these polymers were composed of same monomer units ([-Si(CH_3_)(CH_3_CH_2_CF_3_)O-]_n_). On the basis of their different retention times in GC-MS measurement and the results of ESI-MS analysis, we speculated that the formed volatiles during the synthesis of PTFPMS-sponge were mainly composed of fluorosilicone short chains with 3-9 monomer units.


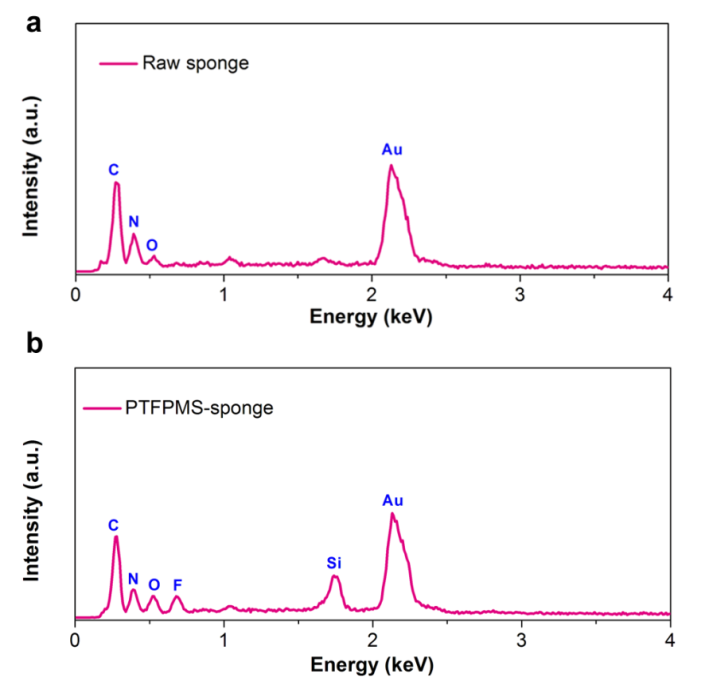


**Figure S4** | EDS spectra showing chemical composition of the sponges: (a) the uncoated MF sponge and (b) PTFPMS-sponge. The peaks of F and Si were clearly observed for PTFPMS-sponge, indicating the successful grafting of PTFPMS on the surface of MF sponge. The signal of Au originates from the sprayed gold.


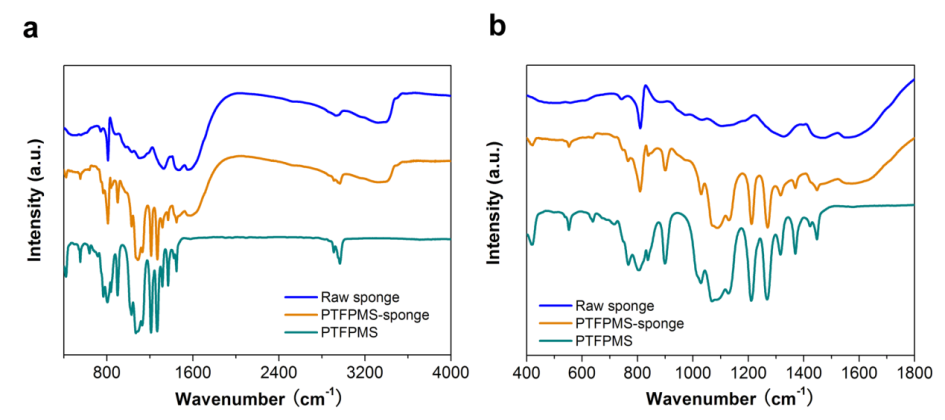


**Figure S5** | FTIR spectra of PTFPMS-sponge, compared with the uncoated MF sponge and original PTFPMS. For PTFPMS-sponge, the peaks located at 1213 and 1315 cm^−1^ are ascribed to characteristic symmetric and asymmetric stretching modes for C-F; the Si-O-Si asymmetric stretching signal was clearly observed from 1027-1129 cm^−1^. The presence of C-F and Si-O-Si signals indicates the successful grafting of PTFPMS on the surface of MF sponge. The peak at 810 cm^−1^ is attributed the characteristic bending vibration of triazine ring in MF sponge. The broad peak at 3100-3400 cm^−1^ can be assigned to the N-H and O-H stretching vibrations of the MF sponge. The IR results suggest that the hydrophobic silicone was successfully coated on the surface of MF sponge, without changing the original composition of the MF sponge.


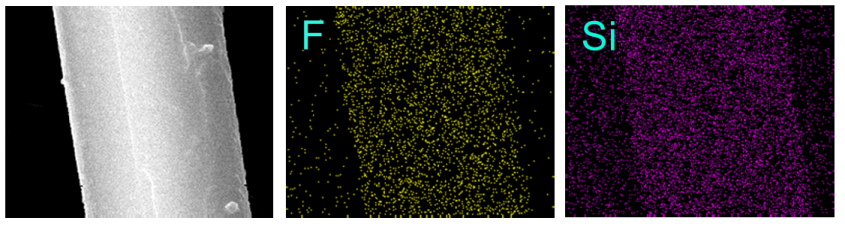


**Figure S6** | Enlarged SEM image and the corresponding elemental mapping images of PTFPMS-sponge, showing the homogeneous distribution of elements F and Si across the fiber.


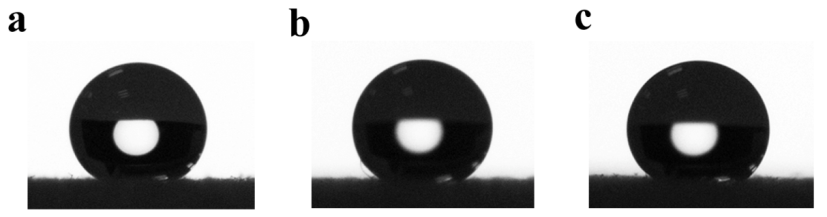


**Figure S7** | The water contact angles of the corresponding MF sponge modified by silicones containing different functional groups: (a) methyl (155.5±3.65º), (b) ethyl (157.1±3.33º), and (c) phenyl (153.3±3.08º).


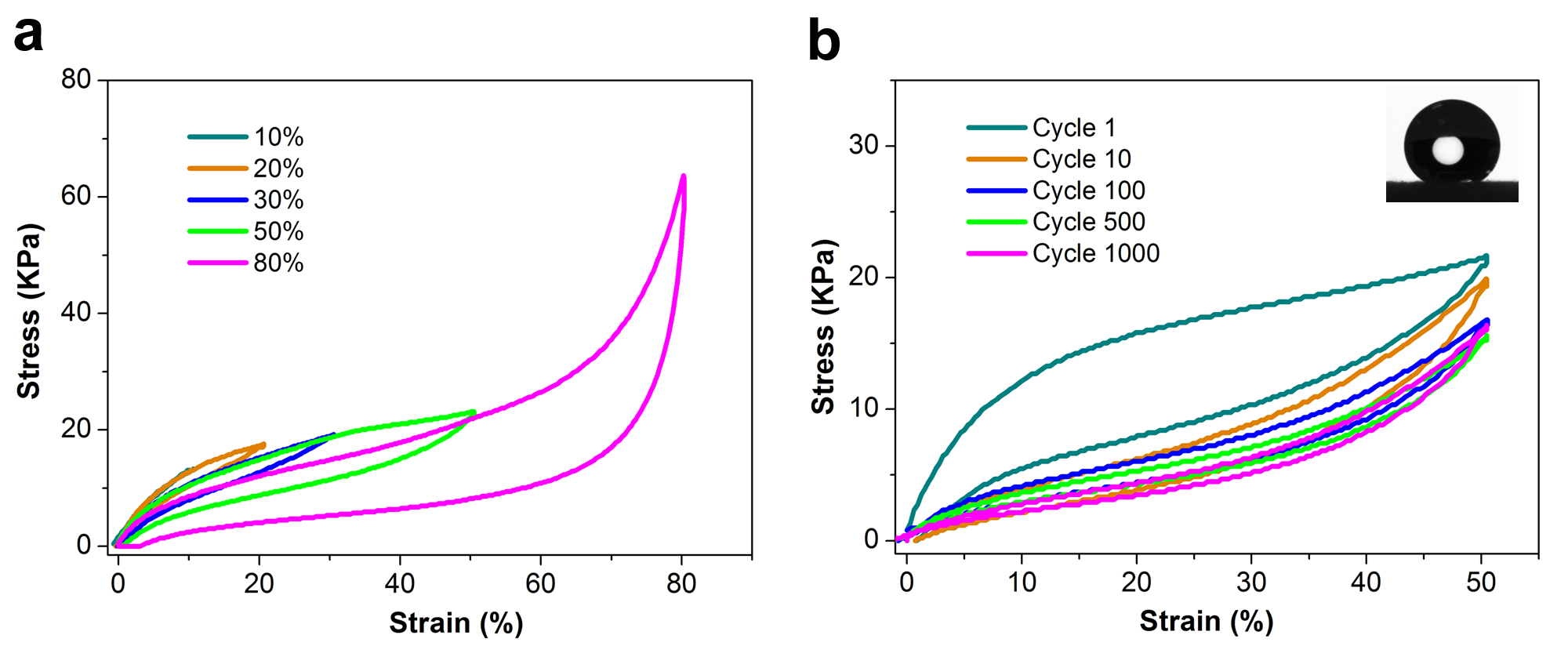


**Figure S8** | (a) The compressive stress-strain curves of PTFPMS-sponge at different strains, the low compressive stress (63.7 kPa at 80% strain) suggests the softness and flexibility of the PTFPMS-sponge; (b) The compressive stress-strain curves of PTFPMS-sponge over 1000 cycles; inset: water contact angle after 1000 cycles of compression test (156.7±2.68º).


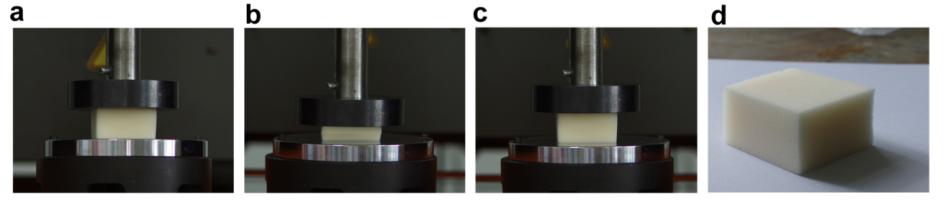


**Figure S9** | (a-c) Sequential photographs of PTFPMS-sponge during the first compression process (one compressing and releasing cycle, 50% strain), indicating the recovery the original shape after the release of compressive stress; (d) photograph of the PTFPMS-sponge after 1000 cycles of compression test (50% strain), the sponge recovers its original shape without obvious structural damage.


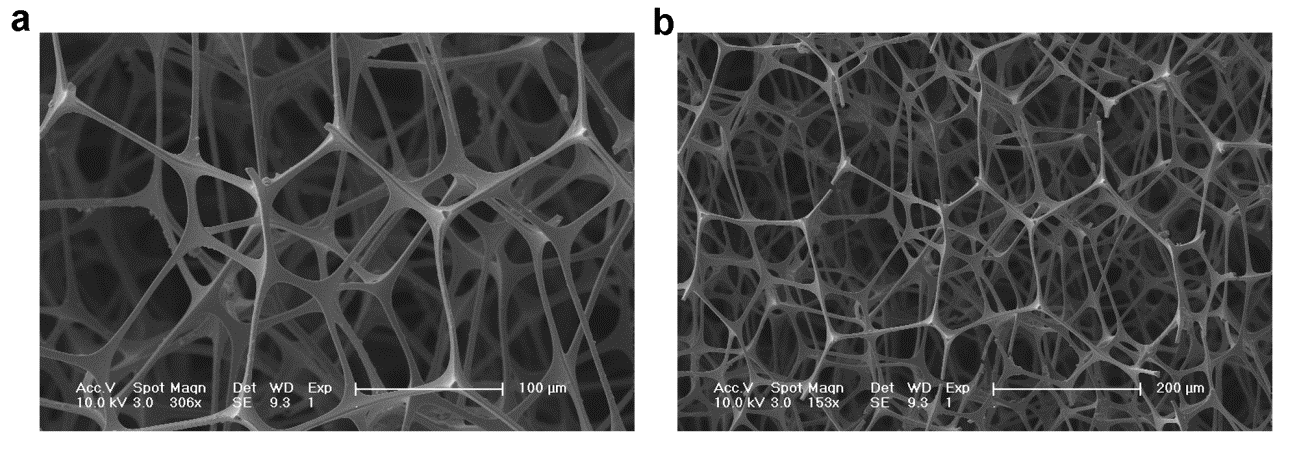


**Figure S10** | SEM images of PTFPMS-sponge after 1000 cycles of compression test (50% strain). The 3D interconnected network and the high porosity of the sponge are maintained, without obvious structural damage, suggesting the robust mechanical stability of PTFPMS-sponge. Such excellent compressive mechanical property will be beneficial to practical applications, particularly for the collection of the absorbed liquids by mechanical squeezing and the recycled usage of the sponge.


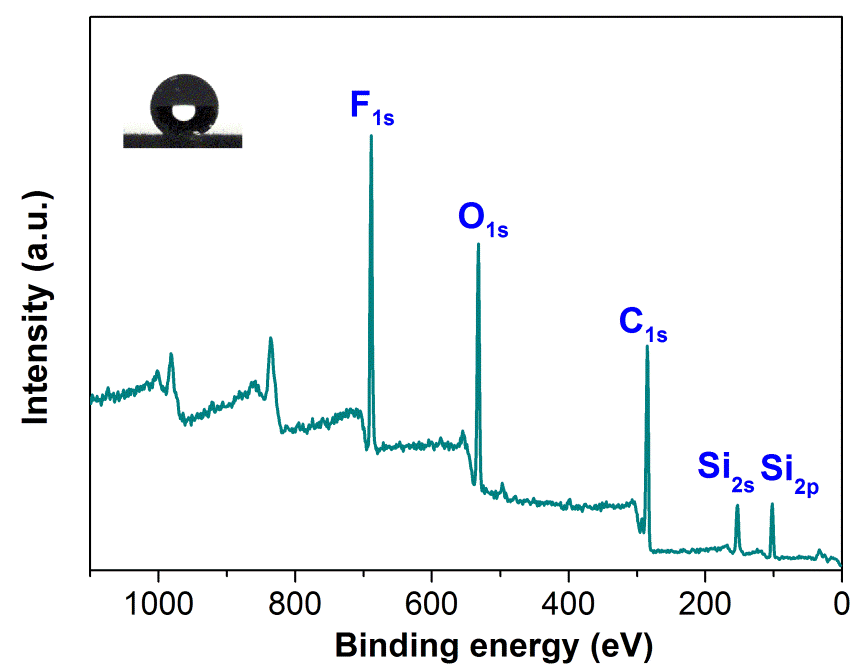


**Figure S11** | XPS survey spectrum and water contact angle (157.0±3.62º) of the PTFPMS-sponge after treated at 250℃ for 1 hour at atmosphere. The high water contact angle and the presence of the signal of F and Si suggested the superhydrophobicity of sponge was well maintained, clearly indicating the superior thermal stability of the hydrophobic coating.


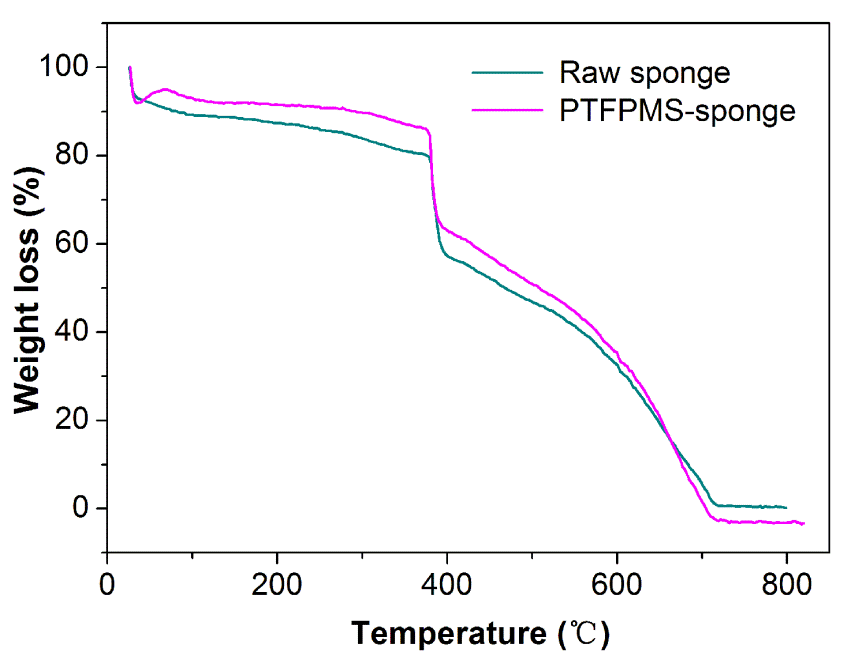


**Figure S12** | Thermogravimetric analysis (TGA) of raw sponge and PTFPMS-sponge, at a heat rate of 10℃ min^-1^, under air atmosphere. Two samples exhibit similar thermal stability. The PTFPMS-sponge is stable up to 376℃, similar to the raw MF sponge. The weight loss below 100℃ should be attributed to desorption of the absorbed solvents.


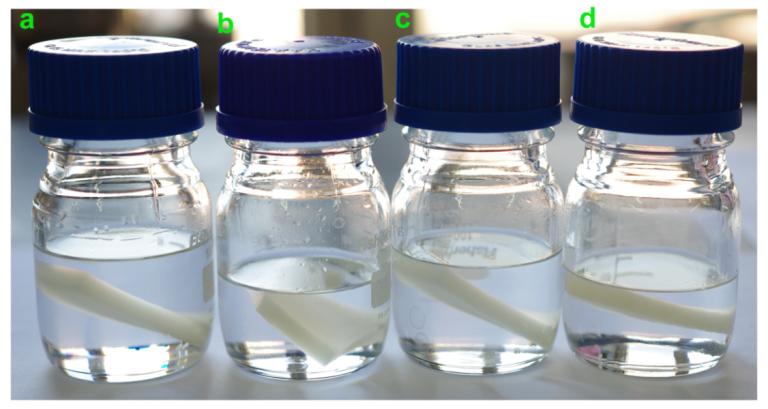


**Figure S13** | Photographs of PTFPMS-sponge after treated with various organic solvents for 12 hours: (a) toluene; (b) cyclohexane; (c) CH_2_Cl_2_ and (d) CHCl_3_.


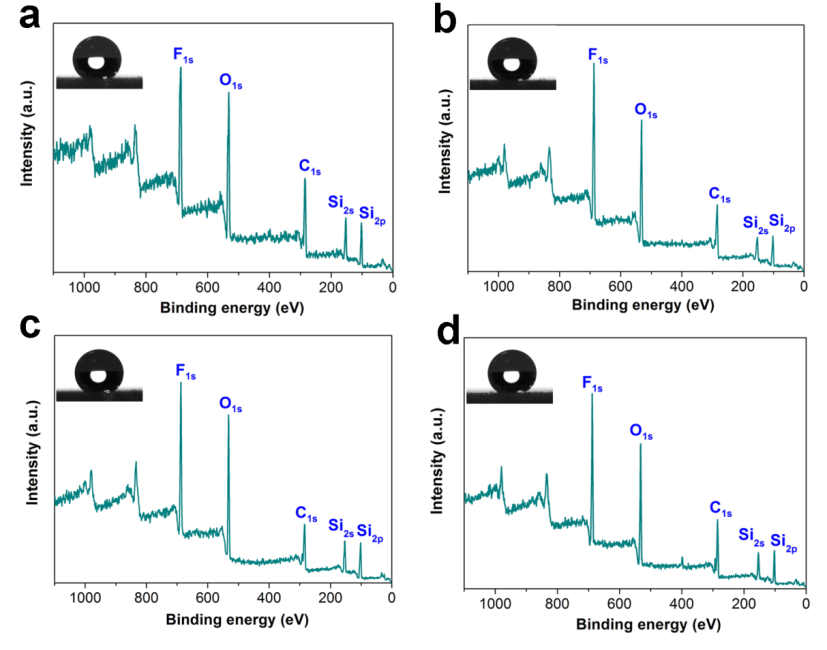


**Figure S14** | XPS survey spectra and water contact angles of the PTFPMS-sponge after treated with various organic solvents for 12 hours: (a) toluene (157.6±1.69º); (b) cyclohexane (154.4±5.24º); (c) CH_2_Cl_2_ (157.2±0.41º) and (d) CHCl_3_ (154.7±3.60º). The signals of F and Si were clearly observed in all the samples, demonstrating the robust stability of the hydrophobic coating. The superhydrophobicity and water-repellent nature of the sponge were well preserved after the immersion test. This will be advantageous for the extensive application of the sponge when clearing various organic compounds and oils.


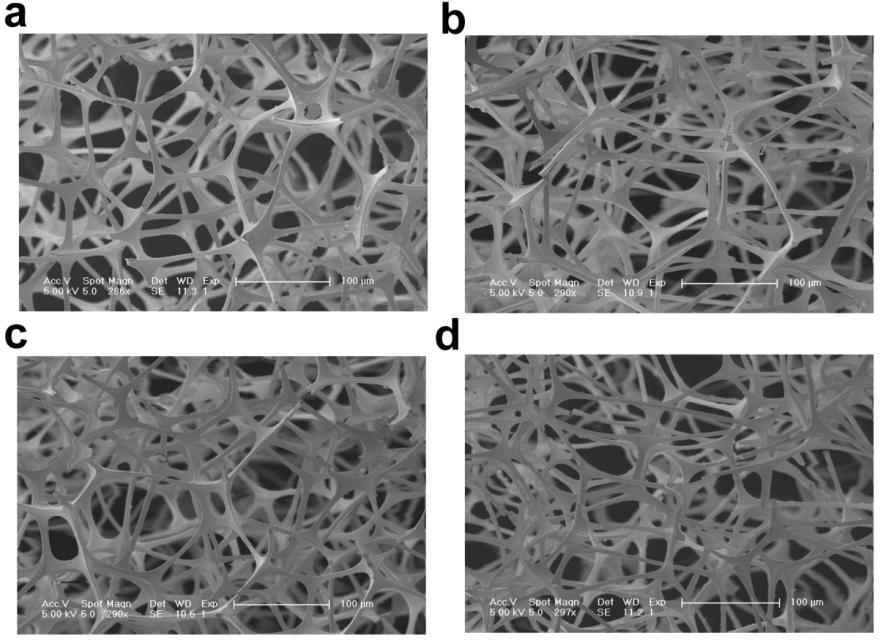


**Figure S15** | SEM images of PTFPMS-sponge after treated with various organic solvents for 12 hours: (a) toluene; (b) cyclohexane; (c) CH_2_Cl_2_ and (d) CHCl_3_. It is clear that the 3D interconnected network and high porosity of the sponge were well maintained after the immersion treatment.


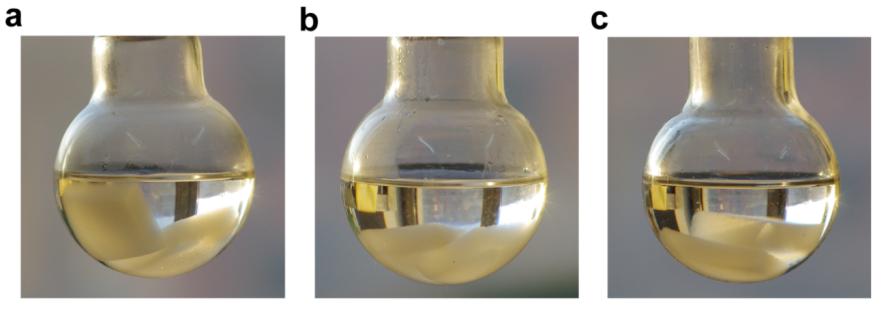


**Figure S16** | Photographs of PTFPMS-sponge after exposure to boiling treatment in: (a) cyclohexane, (b) toluene, and (c) xylene for 1 hour. The robustness of the PTFPMS-sponge was mainly ascribed to the fact that the MF sponge is thermally infusible and resistant to various organic compounds.


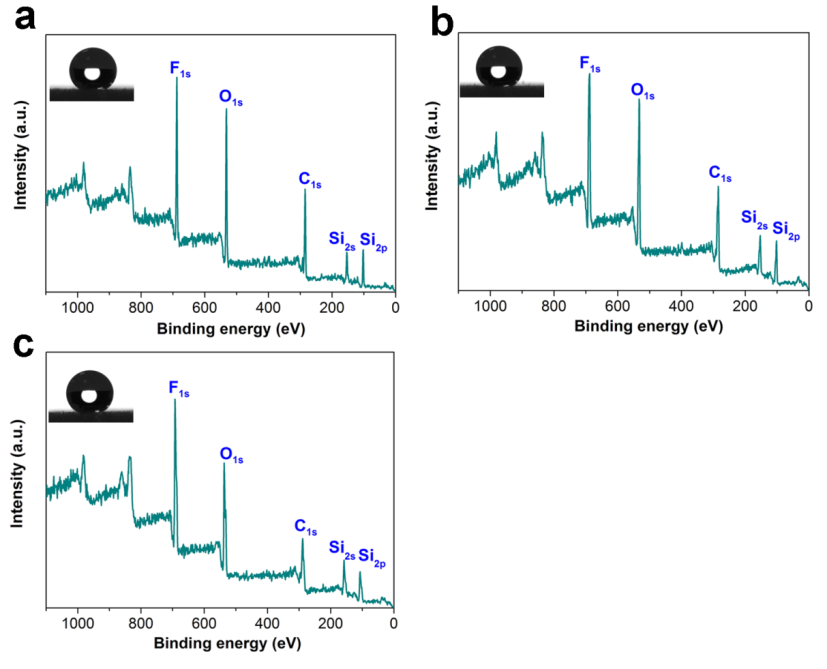


**Figure S17** | XPS survey spectra of and water contact angles of PTFPMS-sponge after exposure to boiling treatment in: (a) cyclohexane (156.2±2.51º), (b) toluene (156.7±0.99º), and (c) xylene (157.2±1.94º) for 1 hour. The signals of F and Si were clearly observed in all the samples, demonstrating the robust stability of the hydrophobic coating. The superhydrophobicity and water-repellent nature of the sponge were well preserved after the boiling treatment. This will be advantageous for the extensive application of the sponge when clearing and recycling various organic compounds and oils.


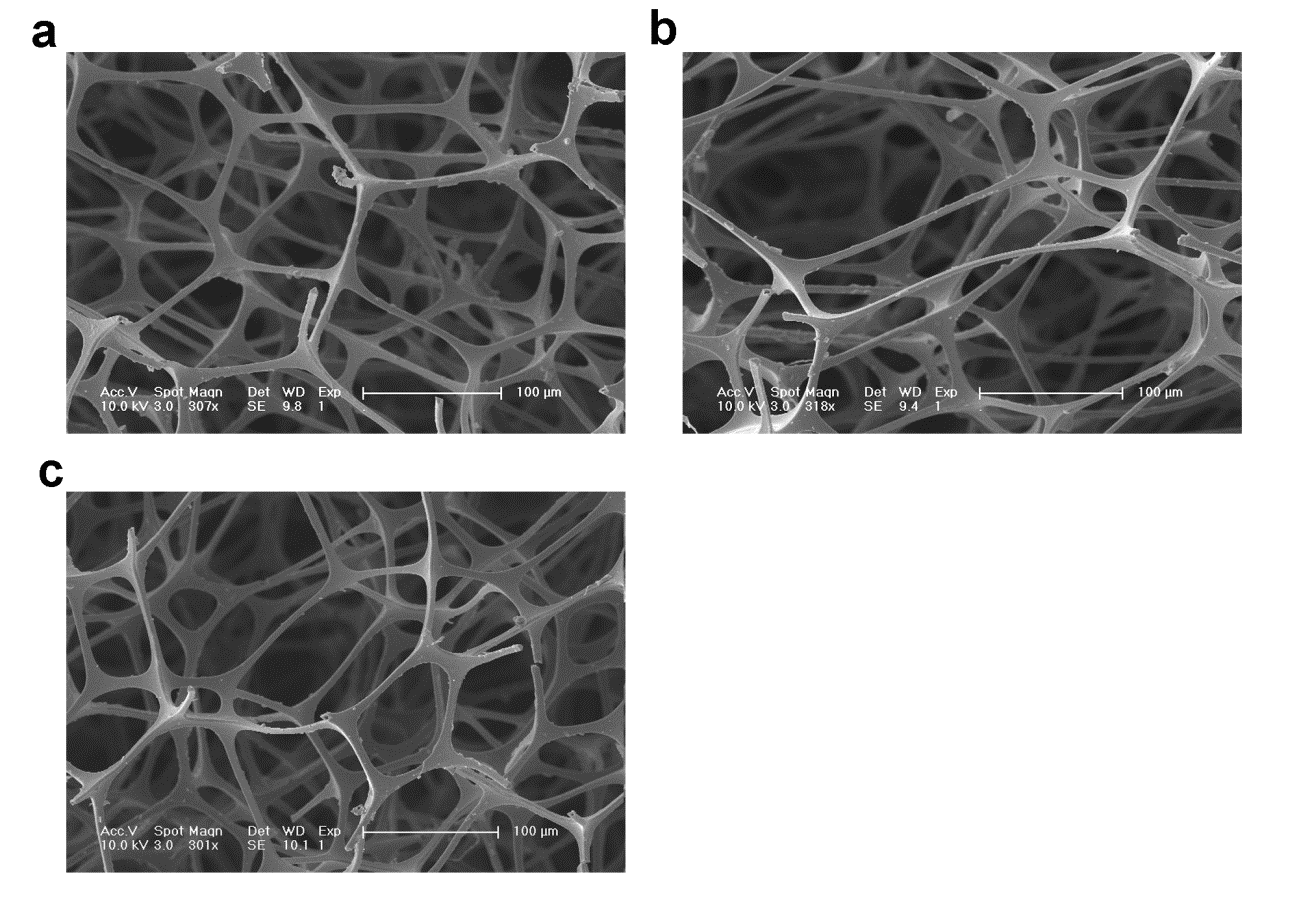


**Figure S18** | SEM images of PTFPMS-sponge after exposure to boiling treatment in: (a) cyclohexane, (b) toluene, and (c) xylene for 1 hour. It is clear that the 3D interconnected network and high porosity of the sponge were well maintained after the boiling treatment.


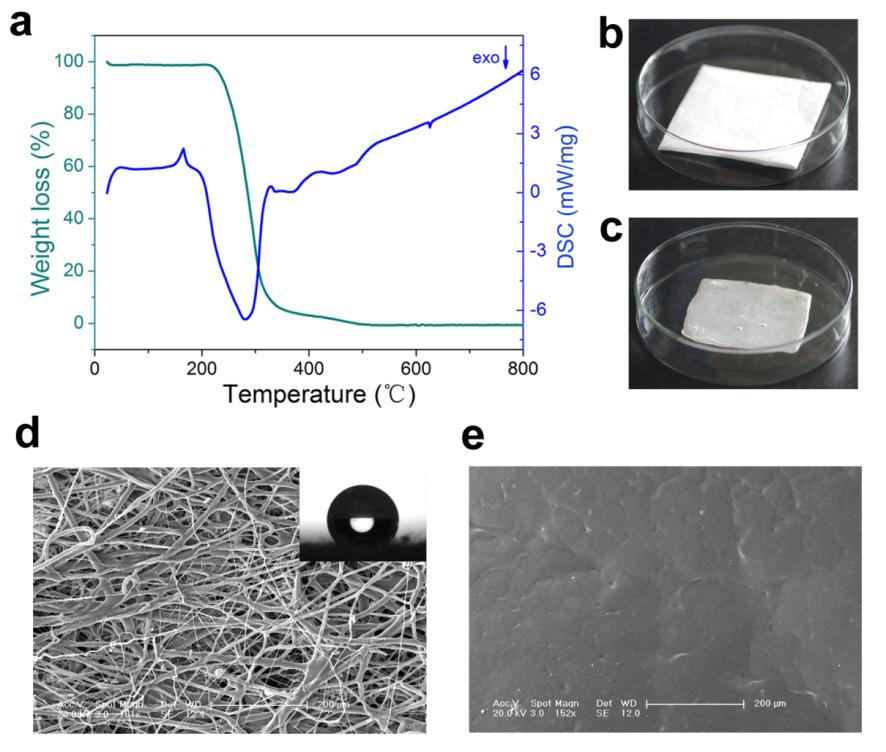


**Figure S19** | Thermal stability of the PP fabric: (a) TGA and DSC of PP fabric, revealing its low melting point (166℃); (b) photograph of a commercial PP fabric (c) photograph of the PP fabric after being heated at 250℃ less than 2 minutes; (d) SEM image of the original PP fabric, inset: water contact angle of the PP fabric (136.7±2.65º); (e) SEM image of the melted PP fabric, indicating the loss of the porosity.


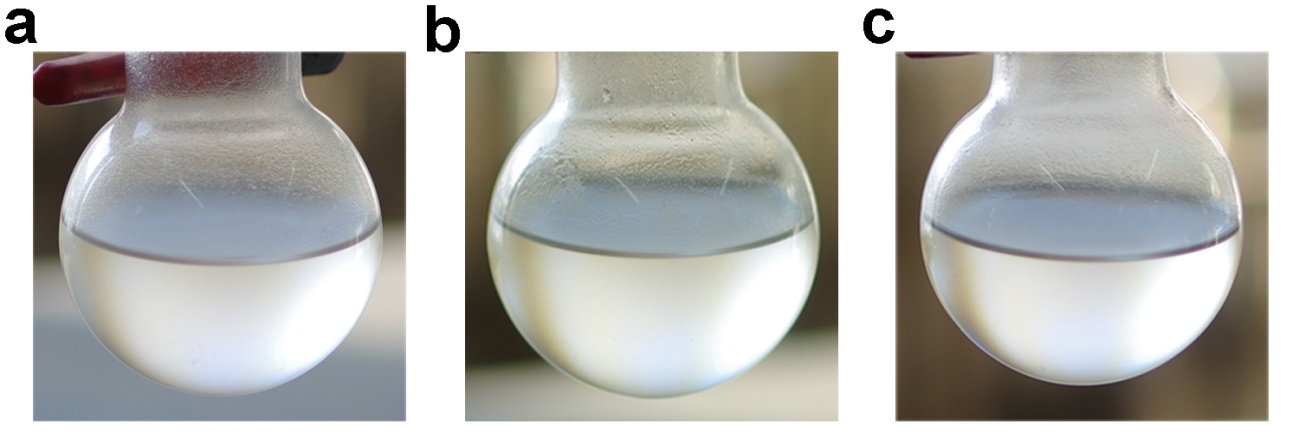


**Figure S20** | Photographs of PP fabric after exposure to boiling treatment in: (a) cyclohexane, (b) toluene, and (c) xylene (1 hour in cyclohexane, less than 10 minutes in toluene and xylene). It is obvious that PP fabric loses its structural integrity, indicating its poor solvent-tolerance to these solvents.


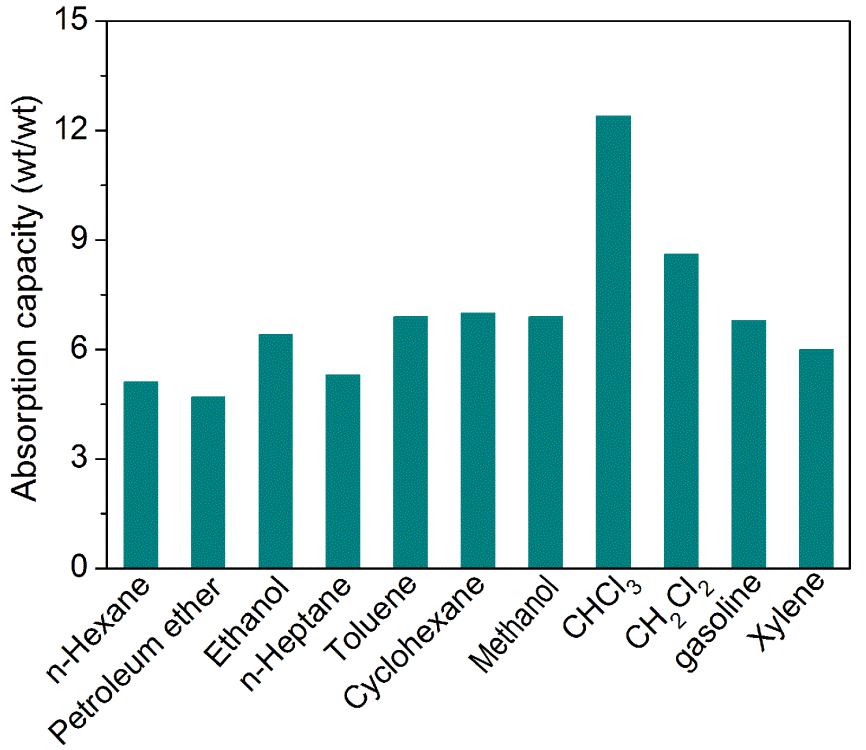


**Figure S21** | Gravimetric absorption capacities of PP fabric for various oils and organic solvents.


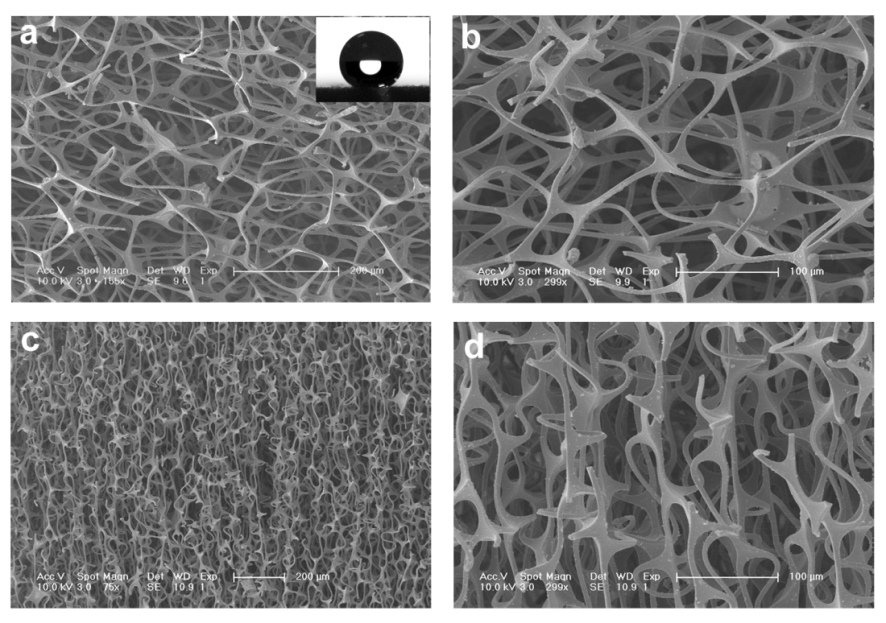


**Figure S2****2** | SEM images of PTFPMS-coated MF membrane: (a-b) front view; (c-d) cross-sectional view. SEM images (both front and cross-sectional view) displayed the 3D interconnected network and highly porous structure of this membrane, which will be advantageous for the fast mass transfer. Inset in (a): the corresponding water contact angle of PTFPMS-coated MF membrane (156.7±2.79º), suggesting the superhydrophobicity and water-repellent nature of the membrane.
